# Supplementary material for: Polycyclic Aromatic Hydrocarbons Activate the Aryl Hydrocarbon Receptor and the Constitutive Androstane Receptor to Regulate Xenobiotic Metabolism in Human Liver Cells
Source: Int J Mol Sci. 2020 Dec 31;22(1):372. doi: 10.3390/ijms22010372 (PMC7796163; doi:10.3390/ijms22010372)
Supplement: Supplementary file 1 [file ijms-22-00372-s001.zip › ijms-1036032 suppl/Supplementary_Plasmids and Primers.pdf]

Supplementary material:

Polycyclic aromatic hydrocarbons activate the aryl hydrocarbon receptor and the constitutive androstane receptor to regulate xenobiotic metabolism in human liver cells

Lisa Goedtke[1], Heike Sprenger[1], Ute Hofmann[2], Felix F. Schmidt[3], Helen S. Hammer[3], Ulrich M. Zanger[2], Oliver Poetz[3], Albrecht Seidel[4], Albert Braeuning[1], Stefanie Hessel-Pras[1]

[1] German Federal Institute for Risk Assessment (BfR), Max-Dohrn-Straße 8-10, 10589 Berlin, Germany

[2] Dr. Margarete Fischer-Bosch Institute of Clinical Pharmacology, Auerbachstr. 112, 70376 Stuttgart, and University of Tübingen, Tübingen, Germany

[3] SIGNATOPE GmbH, Markwiesenstraße 55, 72770 Reutlingen, Germany

[4] Biochemical Institute for Environmental Carcinogens, Prof. Dr. Gernot Grimmer Foundation, Lurup 4, 22927 Grosshansdorf, Germany

Corresponding author:

Dr. Stefanie Hessel-Pras (ORCID: 0000-0002-6153-0035)

German Federal Institute for Risk Assessment, Max-Dohrn-Straße 8-10, 10589 Berlin, Germany

e-mail: stefanie.hessel-pras@bfr.bund.de

Tel: +49 30 18412 25203

Table S1: Plasmids used for transfection of HepG2 cells.

**reporter gene assay**	**Plasmid**	**plasmid amount [ng/well]**
AHR	pGL4.26-XRE6	40
CAR	pcDNA5-GAL4/DBD-hCAR/LBD(+3aa)<br>pGAL4-(UAS)5-TK-LUC	50<br>40
for both assays	pcDNA3-RLuc	1

Table S2: Primer sequences used for gene expression analyses.

**gene**	**sequence**	
CYP1A1	forward	5´-ACCCTGAAGGTGACAGTTCC-3´
	reverse	5´-TCTTGGAGGTGGCTGAGGTA-3´
CYP1A2	forward	5´-CTTCGCTACCTGCCTAACCC-3´
	reverse	5´-CCCGGACACTGTTCTTGTCA-3´
CYP2B6	forward	5´-TTCGGCGATTCTCTGTGACC-3´
	reverse	5´-ATGAGGGCCCCCTTGGAT-3´
ACTB	forward	5´-CCTTGCACATGCCGGAG-3´
	reverse	5´-GCACAGAGCCTCGCCTT-3´
GAPDH	forward	5´-GTCAAGGCTGAGAACGGGAA-3´
	reverse	5´-AAATGAGCCCCAGCCTTCTC-3´
